# Dr. Darwell on a Peculiar Species of Paralysis

**Published:** 1831-01-01

**Authors:** 


### DR. DARWALL ON A PECULIAR SPECIES OF PARALYSIS.*

Dr. D. has met with a species of paralysis, not evidently depending on cerebral or spinal disease, which, so far as he knows, is not described in medical works. Of the actual cause of the complaint, and the proper treatment, he fears that he can furnish little information.

"The first case which occurred to me was in a washerwoman, who had been accustomed to carry heavy weights upon her arms. The paralysis was confined, at first, to the muscles which raise the os humeri; there was also great emaciation of the deltoid muscle; and she could only move the limb a few inches from the trunk, and this with great difficulty. Nevertheless, she could bend the fore-arm upon the arm, and, to use her own expression, she could do any thing under the elbow. The hand had also its full power. There was not the slightest symptom of affection of the head, nor, indeed, excepting this paralysis, did the patient appear to have the slightest ailment. The treatment instituted did no good, and she gradually lost the whole power of the upper extremities; after which time I lost sight of her, and have since heard nothing of the case.

About the same time, another instance presented itself, in a man who was in a cornfactor's warehouse, and was much accustomed to move large bags of oats, beans, &c. Like the former case, his incapacity was at first confined to the muscles which raise the arm, but gradually involved the whole limb, till, at the present time, seven or eight years from the original attack, he has no use whatever in the superior extremities. In this instance, it was suspected that there was some disease in the origin of the nerves supplying the muscles of the arm, and a seton was inserted in the neck, but without

* Mid. Med. and Surg. Reporter, No. X. Nov. 1830.

any good effect. The muscles continued to waste, and the limb to lose its power. In neither of these cases was any pain experienced.

A short time after I had seen this last case, a man applied to me, for a severe pain in the left deltoid muscle. He also had been accustomed to carry very heavy weights, and latterly experienced great weakness in the limb. As in the former case, the muscle was much wasted, and was very nearly half the size of the other. Considering the pain in this instance to be rheumatic, and confined to one muscle, acupuncturation was recurred to, and three needles were inserted in different parts of the deltoid muscle. The result was most satisfactory; the pain disappeared, and the muscle gradually recovered its bulk. From that time to the present, now seven years, there has been no return of the affection, though he suffers severely from a chronic inflammation of the bronchi."

The next case was that of a girl, eighteen years of age, stout and plethoric. According to her account, she suffered excessive pain for several hours in the left arm and shoulder, which terminated in the total abolition of power in the limb. She was bled and purged, the moxa was applied, and then a seton. This came out or was removed by herself, and another was threatened, but most conveniently the whole of the power of the limb returned with a "strong convulsion of the muscles" just before the expected operation. There can be little question that this was an example of that compound of hysteria and humbug, which a good practitioner will seldom mistake, a bad one almost always. For some time no other cases than the foregoing presented themselves to Dr. Darwall, till last May, when he witnessed two more.

The first patient was a locksmith, and had been suffering from severe pain in the right shoulder for some time. Colchicum was ordered, and in a few days he returned free from pain but utterly unable to raise his arm, the deltoid muscle being exceedingly flabby. Electricity was tried with considerable benefit, and after the third application, he stated that he had fully recovered the use of the limb. He has not been seen since.

The last case is still under treatment, and is exactly the counterpart of the first which is mentioned in the paper, excepting that the left arm only is affected. The loss of power had commenced in the muscles that move the shoulder, and had gradually been extending downwards for four months, but she could still do any thing under the elbow. There was slight pain in the deltoid on pressure, and considerable pain in the shoulder-joint on attempting to raise the arm. The muscles of the arm were all greatly emaciated. She had been accustomed to carry heavy weights. Acupuncturation had no effect, and electricity was tried. At first it prevented the further progress of the complaint, and latterly she has acquired additional strength in the limb, and some increase of bulk in the muscles.

"The circumstance," says Dr. Darwall, "particularly calling for notice in these cases, is the pain preceding the loss of power, and the apparent facility with which, while in this state, the farther progress of affection was prevented. Are we also to regard the fact, of several of these persons having been accustomed to carry heavy weights, as merely accidental; or is there any connexion between the exertion thus required) and the after loss of power, as cause and effect? Again, in what way is the pain, which simulates rheumatism in its character, to be regarded? And in what way does electricity act?

The impression on my mind is, that this is primarily an affection of the nerves supplying the elevating muscles, and that they may have been injured by the straining necessary in raising or carrying heavy weights. I am the rather inclined to this opinion, from having observed a similar wasting of muscles, in a case in which the ligaments of the shoulder-joint had been strained, if not broken, and in which, though actual dislocation did not take place, the os humeri hung at a distance from the scapula; the space between the acromion process and the os humeri, being increased to nearly an inch. Now here it was impossible that much straining of the nerves should not take place, and we know that, without the full influence of the nerves, the muscles cannot act. Dr. Wilson Philip's experiments also prove that secretion is under the superintendence of the nerves; and if secretion, then also nutrition must be concluded to be equally under their superintendence. Farther, supposing that a function be not exercised, diminution of power invariably succeeds, and generally wasting of the organ, which is the instrument of the function. Thus, then, we should first have an affection of nerves producing pain, and impeding the use of the muscles; secondly, the muscles being dependent to a certain extent for their nourishment upon the nervous energy, wasting would ensue, and loss of power. And again, the loss of power necessitating a still farther diminution in the exercise of the organs, would increase the emaciation, till not only the function of the muscles would be annihilated, but their structure almost, if not entirely, would be wasted away. I throw out these hints with considerable diffidence, yet, as in all the cases I have seen, the affection attacked persons who were dependent upon their labour for their subsistence, it is an object of no small importance to ascertain the nature of this disease, and to stop it in its first progress. How far electricity will be found serviceable in these cases generally, remains yet to be ascertained. Its action, however, is that of a stimulant, and though it probably is not the same with the energy afforded by the nerves, it has evidently the power of exciting that energy, and where the structures remain unimpaired, it might be expected to be useful."

The cases are deserving of attention.

